# Platelet-Rich Plasma and Platelet-Rich Fibrin Can Induce Apical Closure More Frequently Than Blood-Clot Revascularization for the Regeneration of Immature Permanent Teeth: A Meta-Analysis of Clinical Efficacy

**DOI:** 10.3389/fbioe.2018.00139

**Published:** 2018-10-11

**Authors:** Peter E. Murray

**Affiliations:** Department of Periodontics, College of Dental Medicine, Nova Southeastern University, Fort Lauderdale, FL, United States

**Keywords:** apical closure, saving immature teeth, regenerative endodontics, dental pulp, revascularization

## Abstract

The purpose of this review was to compare the clinical efficacy of platelet-rich plasma (PRP) and platelet-rich fibrin (PRF), vs. blood clot revascularization (BCR) for the regeneration of immature permanent teeth. A survey of the literature identified 222 cases of immature permanent teeth which had been given PRP, PRF or BCR regeneration treatment with at least a year-long follow-up in 12 different articles. A meta-analysis of the 222 immature permanent teeth after 1 year, were compared to assess the ability of PRP, PRF, and BCR to accomplish apical closure, a periapical lesion healing response, root lengthening, and dentinal wall thickening. The mean success rate for apical closure or reduction after 1 year was: PRP (85.1%) PRF (85.2%), and BCR (58.8%). The mean success rate for root lengthening after 1 year was: BCR (64.1%), PRP (64.2%), and PRF (74.1%). The periapical lesion healing response was 88.9% for BCR, 100% for PRP, and 100% for PRF. Dentinal wall thickening was 100% for BCR, 100% for PRP, and 100% for PRF. Apical closure occurred more frequently following PRP and PRF than with BCR (Fischer test, *P* < 0.0011), for all the other effects the PRP, PRF, and BCR treatments were similarly effective (Fischer tests, *P* > 0.05). In conclusion, the fractured or decayed immature permanent teeth of children and young adults aged 6 years to 28 which have a restorable crown, but thin dentinal walls may be regenerated by using a revascularization procedure which draws blood and stem cells into a disinfected root canal space. Although BCR is most common revascularization method, apical closure may occur more frequently if PRF and PRP are used instead of BCR for the regeneration of immature permanent teeth. The proper use of regenerative procedures can be very successful at the disinfection of bacteria from the periapical region of immature permanent teeth, which helps to heal localized lesions, and avoid the need for complex apical surgery, in addition to regenerating tissues to strengthen the structure of immature teeth, to help prevent tooth fracture and tooth loss.

## Introduction

Endodontic treatments can save millions of caries-diseased and fractured permanent mature teeth, which are restorable. The average success rates for endodontic treatment after 22 months was 99.3% (Hannahan and Eleazer, 2008), the success rate remains above 83.34% after 8 years (Vozza et al., 2013), and can be above 86.02% successful after 10 years (Elemam and Pretty, 2011). However, caries-diseased and traumatized permanent incompletely developed teeth, can have a poor prognosis when treated by conventional root canal therapy (Harlamb, 2016). A common problem with incompletely developed teeth is that the dentinal walls are thin and weak, making them prone to a stress-overload fracture (Lawley et al., 2004). The long-term use of calcium hydroxide as a root canal dressing may increase risk of root fracture (Andreasen et al., 2002). Immature teeth that have open and often divergent apices are not suitable for complete cleaning and obturation with traditional techniques and materials (Faizuddin et al., 2015). Ideally incompletely developed teeth require a “regenerative endodontic procedure” (REP) which can regenerate replacement pulp to mineralize and thicken the thin dentinal walls, thereby, strengthening the structure of the tooth. Strengthening the tooth dentinal walls by REP, could help prevent a subsequent fracture of the tooth (Murray et al., 2007). Although REPs appear to be a promising treatment for permanent immature diseased and injured teeth, they can be problematic: REPs might not always result in complete root formation, and may not completely reduce the chances of root fracture (Bose et al., 2009), the success of REPs need to be investigated to identify how to avoid failures, and increase their rate of success.

REPs began around 1952, when a German dentist called: Dr. B. W. Hermann advocated the use of calcium hydroxide as a dressing for after a vital pulp amputation (Hermann, 1952). Today, many dentists still use calcium hydroxide, mainly for apexification (Gawthaman et al., 2013), although MTA has overtaken calcium hydroxide to become the most popular pulp repair material (Monteiro et al., 2017). Subsequent REPs include the development of guided tissue or bone regeneration procedures and distraction osteogenesis (Jani, 1975); the application of platelet rich plasma (PRP) for bone augmentation (Kassolis et al., 2000); the use of platelet rich fibrin (PRF) for periodontal wound healing (Powell et al., 2009); and blood clot revascularization (BCR) by a Norwedgian dentist called Dr. Nygaard-Ostby for the regeneration of tissues within the root canals of pulpotimized teeth (Ostby, 1961; Nygaard-Ostby and Hjortdal, 1971). There may be a resistance among clinicians to use PRP and PRF instead of BCR, because PRP and PRF requires a venous blood draw from the arm of the patient at the time of treatment, and this adds time and complexity to the tasks necessary to deliver the dental treatments, in addition to the added cost of the PRP and PRF kits (Dhurat and Sukesh, 2014).

The efficacy of BCR, or PRP or PRF has proved controversial for the regeneration of teeth: A randomized clinical trial which compared PRP, RPF and BCR concluded that BCR was the standard procedure for revascularization of non-vital permanent immature teeth (Shivashankar et al., 2017). Meanwhile another clinical case series of BCR and PRP, concluded that PRP was the most successful therapy (Jadhav et al., 2013). Another clinical study, concluded that the regenerative effects of PRP and BCR were similar, except that PRP could increase the root length of immature teeth (Alagl et al., 2017). Further research is needed to determine if BCR, or PRP or PRF can give improved treatment outcomes, to guide clinicians to deliver the most effective treatment to benefit patients.

**Figure 1:**
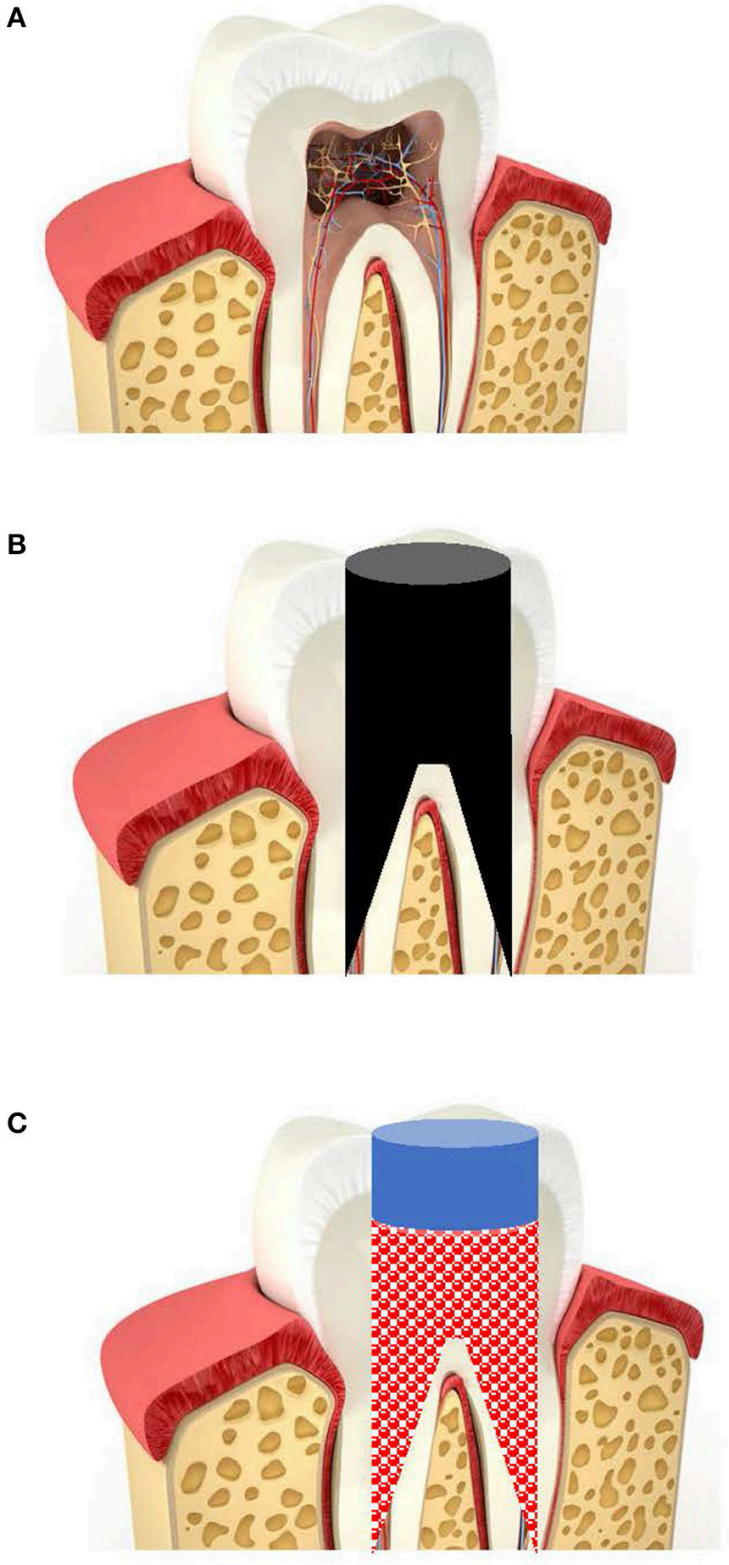
Diagram of the regenerative endodontic treatment of teeth. **(A)** Traumatized or caries-infected immature tooth with a necrotic pulp. **(B)** Removal of necrotic tissues and disinfection of root canal. **(C)** Filling root canals with a BCR with or without PRP or PRF.

The regeneration of teeth with a non-vital necrotic pulp (Figure 1A), requires the removal of the necrotic pulp tissue, to give an empty root canal (Figure 1B), which can then be filled with a BCR, with or without a PRP or PRF scaffold (Figure 1C), populated with viable cells to regenerate replacement pulp tissues to revitalize the tooth.

The successful outcome of the REP can be measured as; the ability to accomplish apical closure of the tooth root, a periapical lesion healing response, root lengthening, and dentinal wall thickening, because these indicate the regeneration of tissues (Jadhav et al., 2013; Alagl et al., 2017; Shivashankar et al., 2017).

The aim of this study was to compare the clinical efficacy of platelet-rich plasma (PRP) and platelet-rich fibrin (PRF), vs. blood clot revascularization (BCR) for the regeneration of 222 immature permanent teeth after 1 year, compared using a meta-analysis for their ability to accomplish apical closure, a periapical lesion healing response, root lengthening, and dentinal wall thickening.

## Materials and methods

The clinical efficacy of PRP, PRF, and BCR to regenerate 222 immature permanent teeth after 1 year from twelve articles (Bose et al., 2009; Jeeruphan et al., 2012; Jadhav et al., 2013; Keswani and Pandey, 2013; Kahler et al., 2014; Bezgin et al., 2015; Tabatabayi et al., 2015; Nagaveni et al., 2016; Alagl et al., 2017; Bakhtiar et al., 2017; Lin et al., 2017; Shivashankar et al., 2017), were compared using a meta-analysis for their ability to accomplish apical closure, a periapical lesion healing response, root lengthening, and dentinal wall thickening.

This study did not require ethical approval from the Institutional Review Board because it used a meta-analysis of a publically-available dataset. The de-identified data was previously published in 12 articles selected from Medline following the search terms “clinical trial, platelet rich plasma/blood clot revascularization/platelet-rich fibrin, or immature teeth.”

The clinical criteria selected for evaluating the success of PRP, PRF, and BCR was apical closure of the tooth root, a periapical lesion healing response, root lengthening, and dentinal wall thickening (Jadhav et al., 2013; Alagl et al., 2017; Shivashankar et al., 2017).

The raw data was analyzed using Chi-Square statistical tests (STATview, SAS, Cary GA, US) for each individual variable: (apical closure of the tooth root, a periapical lesion healing response, root lengthening, and dentinal wall thickening) for each comparison of the procedures (PRP, PRF, and BCR) at a significance level of *P* < 0.05.

## Results

The mean success rate for apical closure or reduction after 1 year was: PRP (85.1%) PRF (85.2%), and BCR (58.8%). The mean success rate for root lengthening after 1 year was: BCR (64.1%), PRP (64.2%), and PRF (74.1%). The periapical lesion healing response was 88.9% for BCR, 100% for PRP, and 100% for PRF. Dentinal wall thickening was 100% for BCR, 100% for PRP, and 100% for PRF. Apical closure occurred more frequently following PRP and PRF than with BCR (Fischer test, *P* < 0.0011), for all the other effects the PRP, PRF, and BCR treatments were similarly effective (Fischer tests, *P* > 0.05).

Dentinal wall thickening was 100% for all the three procedures, which were similarly effective (*P* > 0.05). The individual and mean results for the PRP, PRF, and BCR procedures are summarized in Table 1.

**Table 1:** Meta-analysis data of BCR, PRP, and PRF for the regenerative endodontic treatment of immature teeth following trauma after more than a 1 year follow-up.

**No**.	**Article**	**Procedure (PRP, PRF or BCR)?**	**Apical closure or decrease reported ?**	**Periapical lesion healing response**	**Root lengthening (N)**	**Root lengthening (%)**	**Dentinal wall thickening (N)**	**Dentinal wall thickening (increase in root width %)**
1	Alagl et al., 2017	PRP (*N* = 15)	*N* = 14/15 (93.3%) (Complete closure)	*N* = 15/15 (100%) (All lesions healed)	NA	NA	NA	NA
2	Alagl et al., 2017	BCR (*N* = 15)	*N* = 8/15 (53.3%) (Complete closure)	*N* = 15/15 (100%) (All lesions healed)	NA	NA	NA	NA
3	Bakhtiar et al., 2017	PRF (*N* = 4)	*N* = 4/4 (100%) (Complete closure)	*N* = 4/4 (100%) (Complete healing)	*N* = 4/4 (100%) (Root development)	NA	NA	NA
4	Bezgin et al., 2015	PRP (*N* = 10)	*N* = 7/10 (70%) (Complete closure)	*N* = 7/7 (100% Complete healing)	NA (RRA increased by 9.86%)	NA (RRA increased by 9.86%)	NA	NA
5	Bezgin et al., 2015	BCR (*N* = 10)	*N* = 6/10 (60%) (Complete closure)	*N* = 8/9 (88.9% Complete healing)	NA (RRA increased by 12.6%)	NA (RRA increased by 12.6%)	NA	NA
6	Bose et al., 2009	BCR (*N* = 48)	NA	NA	NA	*N* = 48 teeth analyzed (~10% antibiotic paste) (~ up to 50% after 36 months with Calcium hydroxide)	NA	*N* = 48 teeth analyzed (53.8% (Calcium hydroxide and antibiotic paste after 36 months)
7	Jadhav et al., 2013	PRP (*N* = 3)	*N* = 3/3 (100%) Satisfactory, good and excellent)	*N* = 3/3 (100%) (Satisfactory, good and excellent)	*N* = 3/3 (100%) (Satisfactory, good and excellent)	NA	*N* = 3/3 (100%) (Satisfactory, good and excellent)	NA
8	Jadhav et al., 2013	BCR (*N* = 3)	*N* = 3/3 (100%) (Satisfactory, good and excellent)	*N* = 3/3 (100%) (Satisfactory, good and excellent)	*N* = 3/3 (100%) (Satisfactory, good and excellent)	NA	*N* = 3/3 (100%) (Satisfactory, good and excellent)	NA
9	Jeeruphan et al., 2012	BCR (*N* = 20)	NA	*N* = 16/20 (80% Complete healing)	NA	14.9% (antibiotic paste)		28.2% (antibiotic paste)
10	Kahler et al., 2014	BCR (*N* = 16)	*N* = 3/16 (19.4%) (Complete closure)	*N* = 14/16 (90.3%) (Resolution of radiolucency)	NA	Mean increase 11.3% (−2.7%−25.3%)	NA	Mean increase 35.35% (−1.9%−72.6%)
11	Keswani and Pandey, 2013	PRF (*N* = 1)	*N* = 1/1 (100%) (Complete closure)	*N* = 1/1 (100%) (Complete healing)	*N* = 1/1 (100%) (Root development)	NA	*N* = 1/1 (100%) (Wall thickening)	NA
12	Lin et al., 2017	BCR (*N* = 21)	*N* = 13/21 (61.9% of traumatic teeth following BCR apex decreased (Types 1 and III)	NA	*N* = 9/21 (61.9% of traumatic teeth following BCR root length increased (Types 1 and II)	NA	NA	NA
13	Nagaveni et al., 2016	PRF (*N* = 1)	*N* = 1/1 (100%) (Complete closure)	*N* = 1/1 (100%) (Complete healing)	*N* = 1/1 (100%) (Root development)	NA	*N* = 1/1 (100%) (Wall thickening)	NA
14	Shivashankar et al., 2017	PRF (*N* = 20)	*N* = 16/20 (80%) (Apical foramen response vs. no response)	NA	*N* = 13/20 (65%) (Figure 11)	NA	NA	NA
15	Shivashankar et al., 2017	BCR (*N* = 15)	*N* = 14/15 (93.3%) (Apical foramen response vs. no response)	NA	*N* = 13/15 (86.7%) (Figure 11)	NA	NA	NA
16	Shivashankar et al., 2017	PRP (*N* = 19)	*N* = 16/19 (84.2%) (Apical foramen response vs. no response)	NA	*N* = 12/19 (73.7%) (Figure 11)	NA	NA	NA
17	Tabatabayi et al., 2015	PRF (*N* = 1)	*N* = 1/1 (100%) (Complete closure)	*N* = 1/1 (100%) (Complete healing)	*N* = 1/1 (100%) (Root development)	NA	NA	NA
18	Mean	PRF (*N* = 27)	85.2% (*N* = 23/27)	100% (*N* = 7/7)	74% (*N* = 20/27)	NA	100% (*N* = 2/2)	NA
19	Mean	BCR (*N* = 148)	58.8% (*N* = 47/80)	88.9% (*N* = 56/63)	64.1% (*N* = 25/39)	11.3–50%	100% (*N* = 3/3)	28–53%
20	Mean	PRP (*N* = 47)	85.1% (*N* = 40/47)	100% (*N* = 25/25)	68.2% *N* = 15/22	NA	100% (*N* = 3/3)	NA

## Discussion

Traumatic injuries to incompletely-developed permanent teeth affect 22% of children (Andreasen and Ravn, 1972). Despite this high demand for RETs to save teeth following trauma and caries, the use of endodontic treatments or RET has proved to be controversial (Murray et al., 2007). A survey found that 55.1% of dentists were unsure whether RETs would be successful (Manguno et al., 2012). The failure to use RETs to save immature traumatized due to a lack of training or confidence in the outcome, may cause millions of children to grow up with missing natural teeth that could have been saved. To change clinician opinions and to gain a wider acceptance of RETs to be used to help save children's traumatized teeth it was necessary to determine if the PRP, PRF, and BCR procedures could make RET more successful or if they were similarly effective.

This present study calculated the mean success rate for apical closure and apical reduction after 1 year was:: PRP (85.1%) PRF (85.2%), and BCR (58.8%). PRF was at least 0.1% to 24.4% more effective at inducing apical closure and apical reduction in comparison to PRF and BCR (*P* < 0.0011). Apical closure is a key indicator of immature tooth regeneration (Jadhav et al., 2013; Alagl et al., 2017; Shivashankar et al., 2017). Clinicians who treat hundreds of traumatized immature teeth with REPs, may find that using PRP and PRF will slightly increase the rate of procedure success. Potential disadvantages of the use of PRP and PRF vs. BCR is the increased cost and time needed to prepare the PRP and PRF. Clearly, a child who has trypanophobia (a fear of needles) or hemophobia (a fear of the sight of blood) and do not allow venous blood to be drawn from their arm is not a suitable candidate for PRP or PRF procedures.

The mean success rate for root lengthening after 1 year in this present study was: BCR (64.1%), PRP (64.2%), and PRF (74.1%). Although, PRF was at least 9.9% more effective at inducing root lengthening in comparison to BCR and PRP, the difference was not significantly different in the 222 cases examined here. This data prediction about the superior effectiveness of PRF, supports the theory that blood revascularization through the root apex brings mesenchymal stem cells into the root canal space to help accomplish dentinal regeneration (Lovelace et al., 2011).

The periapical lesion healing response was 88.9% for BCR and 100% for both PRP and PRF. These findings are very good news for patients. It suggests that REPs are very successful at the disinfection of bacteria from the periapical region, which helps to heal loalized lesions, and avoid the need for complex apical surgery (Pinto et al., 2017).

An essential requirement for REPs is to accomplish dentinal wall thickening to strengthen the immature teeth and to help prevent them from suffering a fracture. The amount of remaining tooth structure is the most critical factor for the fracture resistance of endodontically treated teeth (Arunpraditkul et al., 2009). Immature teeth can have very thin dentin walls. The thicker the dentin wall, the less likely that the tooth will fracture, once the thickness exceeds 1.5 mm, the tooth will have an improved fracture resistance (Haralur et al., 2016). Only six cases measured the dentinal wall thickness, and no difference was found between the use of PRP and BCR procedure, which were similarly effective.

In conclusion, the published clinical results for PRP, PRF, and BCR indicate that these treatments are similarly effective for the regeneration of immature permanent teeth. The fractured or decayed immature permanent teeth of children and young adults aged 6 years to 28 which have a restorable crown, but thin dentinal walls may be regenerated by using a revascularization procedure which draws blood and stem cells into a disinfected root canal space. This study has shown that in addition to the most common method of using a BCR technique, a PRP and PRF technique may also be used as alternatives. A drawback of the PRP and PRF techniques is extra time needed to draw blood and centrifuge it prior to insertion into the root canals. The proper use of regenerative procedures can be very successful at the disinfection of bacteria from the periapical region of immature permanent teeth, which helps to heal loalized lesions, and avoid the need for complex apical surgery, in addition to regenerating tissues to strengthen the structure of immature teeth, to help prevent tooth fracture and tooth loss.

## Author contributions

The author confirms being the sole contributor of this work and approved it for publication.

### Conflict of interest statement

The author declares that the research was conducted in the absence of any commercial or financial relationships that could be construed as a potential conflict of interest.
